# Rapid-2-rapid – a collaborative approach to linkage for those recently infected

**DOI:** 10.1186/1471-2334-14-S2-P22

**Published:** 2014-05-23

**Authors:** Eugene Martin, Gratian Salaru, Joanne Corbo, Loretta Dutton, Linda Berezny, Steven Saunders, Sindy Paul, Evan Cadoff

**Affiliations:** 1 Rutgers University - Robert Wood Johnson Medical School, Somerset, New Jersey, USA

## Introduction

In light of evolving, 4th generation HIV diagnostics, more effective strategies to test, link, and retain clients in care, are needed. In New Jersey, USA medical facilities are twice as effective at engaging clients into HIV care as alternative screening entities including community based and public health screening facilities. The goal: Complete screening, confirmation, linkage and entry into care within 2 business days.

## Methods

A facilitated, collaborative process that allows rapid engagement and treatment for all including those at highest risk of infecting others has been developed. Seven collaborations have been established across the State of NJ to connect HIV screening facilities with medical partners. Entry into care is facilitated by Patient Navigators. A two-test, sequential rapid test algorithm (RTA) is used to expedite initial screen confirmation and linkage into care improving the percentage of positive clients linked into care. A community-based screening site performs the first test; the collaborating medical site performs a second orthogonal test. The intake facility has credible verification and immediate justification for laboratory intake. The Patient Navigator serves as a concierge, tester, and facilitator.

## Results

To date, 165,317 ‘rapid-rapid’ tests have been performed in NJ. Data to be discussed includes performance data on the multi-facility orthogonal process, entry and linkage into care. Clients testing positive in rapid-rapid sites are characterized as: NEW POSITIVES, RE-ENGAGED clients or as ALREADY IN CARE.

## Conclusions

Rapid screening with 4th generation HIV diagnostics coupled with rapid identification of acute HIV infection permits rapid initiation of treatment and the potential to reduce transmission. Collaboration between screening facilities and medical facilities provides expedited entry into care and permits treatment within 2 days across a system of more than 30 facilities.

**Table 1:** 

	NEW POS	RE-ENGAGED	ALREADY IN-Care	CLIENT REFUSED	Denied Chanty Care (ID)	Avg. Bus Days to Complete Linkage and Lab Intake	Appt No Show
YTD (Through November, 2012)	131	55	9	12	4	1.48	26

